# An improved ant colony algorithm with diversified solutions based on the immune strategy

**DOI:** 10.1186/1471-2105-7-S4-S3

**Published:** 2006-12-12

**Authors:** Ling Qin, Yi Pan, Ling Chen, Yixin Chen

**Affiliations:** 1 Department of Computer Science, Nanjing University of Aeronautics and Astronautics, Nanjing, 210096, China; 2 Department of Computer Science, Georgia State University, 34 Peachtree Street, Suite 1450, Atlanta, GA 30302-4110, USA; 3 Department of Computer Science, Yangzhou University, Yangzhou, 225009, China; 4 Department of Computer Science and Engineering, Washington University in St. Louis, St Louis, MO 63130, USA

## Abstract

**Background:**

Ant colony algorithm has emerged recently as a new meta-heuristic method, which is inspired from the behaviours of real ants for solving NP-hard problems. However, the classical ant colony algorithm also has its defects of stagnation and premature. This paper aims at remedying these problems.

**Results:**

In this paper, we propose an adaptive ant colony algorithm that simulates the behaviour of biological immune system. The solutions of the problem are much more diversified than traditional ant colony algorithms.

**Conclusion:**

The proposed method for improving the performance of traditional ant colony algorithm takes into account the polarization of the colonies, and adaptively adjusts the distribution of the solutions obtained by the ants. This makes the solutions more diverse so as to avoid the stagnation and premature phenomena.

## Background

Ant colony algorithm (AC) belongs to the class of problem-solving strategies derived from nature. Dorigo, Maniezzo, and Colorni [1], using the Traveling Salesman Problem (TSP) [2,3] as example, introduced the first AC algorithm. With the further study in this area, ant colony algorithm is widely applied to the problems of Quadratic Assignment Problem (QAP) [4], the Frequence Assignment Problem (FAP) [5], the Sequential Ordering Problem (SOP) and some other NP-complete problems. It demonstrates its superiority of solving complicated combinatorial optimization problems.

AC algorithms have been inspired by colonies of real ants, which deposit a chemical substance called pheromone on the ground. This substance influences the choices they make: the more pheromone is on a particular path, the larger the probability is that an ant selects the path. Artificial ants in AC algorithms behave in a similar way. Thus, these behaviours of ant colony construct a positive feedback loop, and the pheromone is used to communicate information among individuals finding the shortest path from a food source to the nest. Ant colony algorithm simulates this mechanism of optimization, which can find the optimal solutions by means of communications and cooperation with each other.

The essence of the optimization process in ant colony algorithm is that: 1. Learning mechanism: the more trail information an edge has, the more probability of it being selected, 2. Updating mechanism: the intensity of trail information on the edge would be increased by the ants passing it and decreased by evaporation, 3. Cooperative mechanism: the communications and cooperation between individuals by trail information enable the ant colony algorithm to have strong capability of finding the best solutions.

However, the classical ant colony algorithm also has its defects. For instance, since the moving of the ants is stochastic, while the population size is large enough, it would take quite a long time to find a better solution. M. Dorigo et al. improved the classical AC algorithm and proposed a much more general algorithm called "Ant-Q System" [8,9]. This algorithm allows the path which has the most intensive trail information with much higher probability to be selected so as to make full use of the learning mechanism and stronger exploitation of the feedback information of the best solution.

In order to overcome the stagnation behaviour in the Ant-Q System, T. Stutzle et al. presented the MAX-MIN Ant System [9,10] in which the allowed range of the trail information in each edge is limited to a certain interval. Wu Qinhong et al. adopted the mutation mechanism in the ant colony algorithm to overcome the stagnation and to get fast convergence speed at the same time [10]. On the other hand, L. M. Gambardella et al. proposed the Hybrid Ant System (HAS). In each iteration of HAS, local probe is made for each solution constructed by ants was used so as to find the local optimal. The previous solutions of the ants would be replaced by these local optimal solutions so that the quality of the solutions could be improved quickly.

However, pursuing a most quick convergence speed may cause problems such as super individual phenomena. Super individuals refer to the ants which have much higher fitness values than other individuals, they overwhelm the solution set and their components often have high pheromone density. After some iteration, the new generations may be constructed mostly by these super individuals or by the components of them, and this will lead to the termination of the search process of the ant colony, that is to say, the algorithm is trapped into local convergence. These super individuals may reduce the global search capability of the ants, since it can only obtain local optimal solutions, i.e. premature solutions.

The other drawback of the classical ant colony algorithm is the close racing problem. If the fitness values and the structures of the individuals in the solution colony are close or approximate with each other, i.e. the spots corresponding to these individuals in the solution space are closed to each other [1,6], the ants' search ability would be reduced, and the process of finding optimal solutions of the ant colony algorithm may be limited into a close local space, which may lead to the stagnation and close race problem. The major factor causing the two problems mentioned above is its lack of a diversity protection mechanism, which can keep the balance between the convergence speed and the quality of the solutions.

On the other hand, the research of ant colony algorithm at present was mainly focused on the human brain intelligence and its learning mechanism, but these personification methods can hardly take into account another hidden intelligence system named immune system. For the immune system of the organism has a high level of intelligence, and lots of superiorities such as immune selection, immune memory, immune metabolism, density control etc. These mechanisms and design optimization algorithms can be used to solve some complicated problems.

## Results

### Mapping the relationship of antibody and antigen to optimization problems

Antibodies generated in the immune system are usually used to resist or eliminate the antigens, and if it maps this relationship into the optimization problem, looking at the object function as the antigen, moreover, the optimal solution of the given problem will be looked at as the antibody. Meanwhile, the characteristics of the immune system will play an illumination role in improving the performance of the ant colony algorithm. In this paper, we simulate the behaviours of immune recomposition, immune memory, immune selection and density control etc.; design DGAA to solve different kinds of optimization problems.

### Diversifying solutions using immune strategy

**Figure 1:**
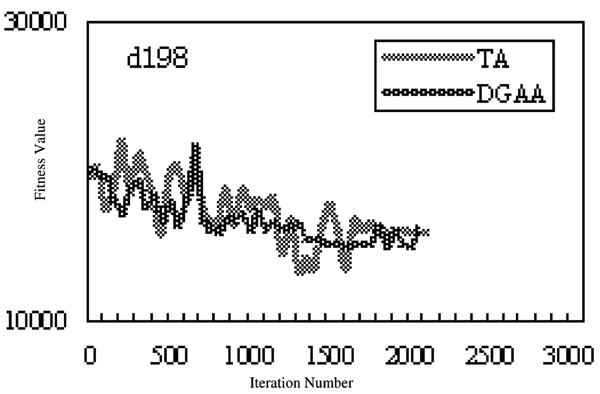
**The evolutionary process for D198**. Fig. 1 and Fig. 2 show the process of the best solution of problems for d198 and ry48p using TA and DGAA respectively. It is obvious that the speed of reaching the best solution using DGAA is higher than that of using TA.

**Figure 2:**
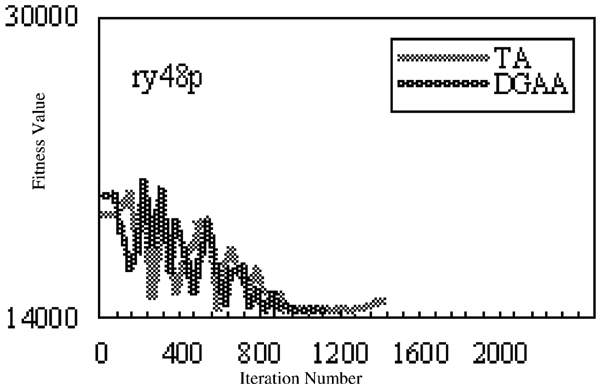
**The evolutionary process for Ry48p**. In DGAA, after reaching the best solution, the curve fluctuates within a very narrow scope around the best solution, this confirms the conclusion that DGAA has higher convergence speed stability and is suitable for solving large scaled TSP problems....

In this algorithm, the individuals are selected to have crossovers and mutation operations according to their fitness value and the diversity of the solutions, and the mutation probability is determined by the diversity of the solutions. At the same time, the isolation niche technique separates the ant colony into several sub-colonies to execute the evolutionary course. In the search process, these sub-colonies are independent with each other and maintain their own features; hence, the algorithm becomes more and more flexible. Experimental results on TSP (Travelling Salesman Problem) showed in Fig. 1, Fig 2 and Table 1, 2 and 3 demonstrate that our algorithm has strong optimization capability; it has diversified solutions, high convergence speed, and succeeds in avoiding the stagnation and premature phenomena.

## Discussions

We want to provide a diversity guaranteed ant colony algorithm (DGAA) by adopting the immunogenic methods to ant colony algorithm and simulating the behaviours of biological immune system. This new type of ant colony algorithm uses the immunogenic methods of immune selection, immune memory, immune metabolism, density controlled strategy and isolation niche technique. But how to integrate immune strategies into ant colony, how to verify its performance is still an open problem for us.

The ant colony algorithm is mainly composed by an iterative algorithm including generation and verification operation, and the global search often realized by the pheromone feedback of the individuals in the colony, so that each individual can has an evolutionary chance or tendency, but meanwhile it may also be degenerated inevitably, even this degeneration is evident under some circumstances.

According to the conception and theory of immunology [11,12], to maintain the eminent performance of the classical algorithm, we make full use of the characteristic of the problems and coalesce the specialty of the immune system to restrain the degeneration in the iteration.

## Conclusion

The method for improving the performance of traditional ant colony algorithm presented here was performed on a Pentium PC. The experiment results showed in Fig. 1 and Fig. 2 was carried out by applying the algorithm to symmetric and asymmetric TSP benchmarks provided by TSPLIB. These TSP benchmarks were also tested using the classical ant colony algorithm to compare its performance with our algorithm.

In DGAA the probability of ants selecting the vertexes and the increment of pheromone updating are adaptively adjusted according to the solutions of the former iteration of the ants. This makes the system never intensify the pheromone on the best path excessively, while the ants can make their evolutionary search towards the correct directions to get strong "mountain climb" capabilities in all directions of the solution space, and obtain optimal solution efficiently.

In addition, DGAA takes into account the polarization of the colonies, and adaptively adjusts the distribution of the solutions obtained by the ants. This makes the solutions more diverse so as to avoid the stagnation and premature. Therefore, our algorithm makes a dynamic balance between convergence speed and stagnation, and this also shows that it is suitable for solving large scaled optimization problems.

## Methods

### Traditional Ant Colony Algorithm

Here we briefly introduce AC and its applications using TSP as an example. In the TSP, a given set of n cities has to be visited exactly once and the tour ends in the initial city. We denote the edge between city i and j as (i, j) and its distance as d_ij _(i, j ∈ [1, n]). Let *τ*_ij_(t) be the intensity of pheromone on (i, j) at time t, and use *τ*_ij_(t) to simulate the pheromone of real ants. Suppose m is the total number of ants, at time t the kth ant selects from its current city i to city j according to the following probability distribution:



Where allowed_k _is a set of the cities can be chosen by the kth ant at city i for the next step, *η*_ij _is a heuristic function which is defined as the visibility of the link between cities i and j, for instance it can defined as 1/d_ij_.

The relative influence of the trail information *τ*_ij_(t) and the visibility *η*_ij _are determined by the parameters *α*, *β*. When *α *= 1 and *β *= 0, the algorithm becomes a complete heuristic algorithm with positive feedback and when *α *= 0 and *β *= 1, it is just a traditional greedy algorithm. For every ant, its path traversing all the cities forms a solution. The intensity of pheromone is updated by Eq. (2):

*τ*_ij_(t + 1) = *ρτ*_ij_(t) + Δ*τ*_ij _    (2)

Where 0 <*ρ *< 1 represents the evaporation of *τ*_ij_(t) between time t and t + 1, Δ*τ*_ij _is the increment of the pheromone on (i, j) in step t, and Δ*τ*_ij_k is the pheromone laid by the kth ant on it, it takes different formula depending on the model used.



For example, in the most popularly used model called "ant circle system", it is given as Eq. (4).



Where Q is a constant, L_k _is the total length of current tour travelled by the kth ant.

### Diversity Guaranteed Ant Colony Algorithm with Immune Strategy

Begin

1. Initialize

The ants search the solution space at random and generate Np initial solutions, compute their fitness, set t = 1(Np is the population size of the colony, t is the iteration number), suppose there are s initial solutions which pass the edge (i, j) and the total length of the paths they have toured are L1, L2,...,Ls, then let the initial trail information on edge (i, j) be



Using niche technique [13], isolate the ant colony to m sub-colonies;

2. While (t <= Nc)

/* Nc is the maximal iteration number depend on the requirement*/

2.1 each sub-colony executes its evolutionary iteration independently

2.1.1 find the ant i which has the most high fitness value f_i _in current sub-colony, decompose its solution it searched, and abstract immune vaccination H = {h_s_|s = 1,2,⋯n, h_s _represents the index number in the solution sequence}

2.1.2 For k = 1 to N do//for n cities

For i = 1 to m do //for m ants

2.1.2.1 The ant i select the next city j with the probability given by (1);

2.1.2.2 have local updating operation to update the trail information on edge (i, j) according to (6);

End for i

The ant would stop its search in current iteration if the length of the path it has passed overpasses that of the optimal solution f_opt _obtained in the former iteration, and then let f_opt _be the solution of this ant;

End for k

2.1.3 Select some solutions searched by the ants in current sub-colony to have mutation operation,

2.1.4 Inoculate the vaccination; have immune memory and immune metabolism operations on the sub-colony;

2.1.5 Calculate the quality factor of each ant, we let *δ *be the density of the ant i, (it was defined in the next section), have global pheromone updating operation to update the trail information on the edges between each city according to (8);

2.2 Adjust the population size of the sub-colony, by eliminating the individuals with lower fitness;

2.3 Have immune memory and immune metabolism operations on the whole colony

End while

3 Return the number of iteration t and the best solution

End

In the line (2.1.2.1) of the algorithm, the next city j will be selected by the following formula:



Here, q is generated stochastically between (0, 1). q_0 _is a probability of pitching on the optimal individual, for example, if q_0 _= 0.7, the city who has the highest trail information will be chosen with a probability over 0.7, meanwhile, the other cities only can be selected with a little chance less than 0.3. In the formula, arg max *τ*_*ij*_(*t*)(*j *∈ *allowed*_*k*_) is the available adjacent city of city i who has the highest trail information. In (5), j_0 _∈ [1, ki] is generated with the probability of *p*_*ij*0_^*k*^(*t*).



### Immune Pheromone Updating Strategy

Suppose the number of total iterations is NC, the fitness of the ith ant at the tth iteration (i.e. at time t) is fit(t, i), which is in inverse proportion to the length of its solution. We denote the worst and best fitness of solutions of the tth iteration as fit_min_(t) and fit_max_(t) respectively, namely f_min_(t) = min{fit(t, i), i ≤ m}, fitmax(t) = max{fit(t, i), i ≤ m}. We define the global worst and best fitness as the worst and best fitness of solutions of all the iterations so far, and denote them as GF_min _and GF_max_, i.e. GF_min _= min{fit_min_(t), t ≤ NC}, GF_max _= max{fit_max_(t), t ≤ NC}. Then the quality factor of ith ant's solution is defined as:



Here, fit(t, i)-GF_min _is the difference between the ith ant's fitness on the current iteration and the global worst fitness. For fixed values of GF_min _and GF_max_, the lager fit(t, i) is, the larger the quality factor the ith ant has.

In our algorithm, we present an adaptive pheromone updating strategy based on quality factor. In line 2.1.2.2, the local updating method is based on (8)





Here length is the length of the solution of the ith ant in the current iteration. By the quality factor, the tour of the ant with the high fitness will be greatly increased to strengthen the pheromone on the tour of the best solution.

### Isolation Niche Technique

In biology, niche means a special role or function in certain circumstances, and organizations that have common organizational functions in given environment are called species. The niche in nature makes the form of new species come true, and this is one of the foundational reasons of the infinity and diversity of the species. Researches have reported that, the creature can come into being daedal species in their evolutionary process mainly due to their capability of separating the whole colony into smaller sub-colonies. In the course of separation, geological isolation plays an important role, it gradually generate biological and behavioural isolation.

There is no communication between separated isolation sub-colonies, and the individuals belonging to different sub-colonies may have distinctly different evolutionary directions and arise different mutation, which may bring bigger and bigger differences.

To multi-peak object function optimization problems, the most important thing is not only getting diversified solutions but also to avoid local optimal solution in the evolutionary process to exploit new search space. Therefore, we can do nothing but construct a new algorithm with potential evolutionary capability to find the optimal solution of the object function. And this new algorithm will apply isolation niche technique into the ant colony algorithm to improve its optimization ability.

In line 1.2 the algorithm implements initial isolation operation. Np initial individuals are assigned to m sub-colonies (i.e. m species), and the number of individuals in each sub-colony is Np/m.

In line 2.2, the population size of the sub-colony is defined according to its average fitness value. The higher average fitness value the sub-colony has, the more chance of propagation this species has, and the larger number of ants the sub-colony will have. But in the ant colony algorithm the population size of the whole ant colony M is fixed (it is often close to the scale of the problem n). In order to avoid unbounded increase of the sub-colony size, we set the maximum size of the sub-colony be s_max_. Contrarily, the number of species may be decreased even be extinct due to the low fitness of some individuals.

In the isolation niche mechanism of this paper, for the sake of protecting some low fitness valued sub-colonies especially to make it survival from the competition and maintaining the evolutionary capability, i.e. maintain the population size of the whole colony, we also define a minimal size s_min _for each sub-colony.

In line 2.3, immune memory operation was carried out on the sub-colonies. Usually, the best individual in current iteration may necessarily have a high probability of coming into being the optimal solution of the object problem. So, we use the immune memory function of the immune system, and copy the sub-colony where the best individual included to the next iteration directly.

In addition, if the sub-colony has low fitness values continually in consecutive iterations, we will perform the immune metabolism operation and generate a new sub-colony in the search space to replace this sub-colony artificially. In the algorithm, some new generated sub-colonies are in the initial stages of its evolutionary process, they are relatively weak compared with other developed sub-colonies although they have potential of evolutionary capability. Therefore, our algorithm will protect the new sub-colonies from elimination in the iterations of its initial stages.

### Vaccination Inoculation

Line 2.1.4 of the algorithm inoculates the vaccination to some selected individuals. It picks out a certain number of ants from current ant colony to inoculate vaccination with a probability of p_v_, then crossover the solution with the vaccination. In the operation of vaccination inoculation, we adopt the order crossover method (OX) proposed by Davis in 1985 [21].

Suppose the paternal individuals to be:

p1 = (2 5 8 3 7 4 6 1)

p2 = (5 1 2 8 3 6 7 4)

One of the paternal individuals is the selected ant to be inoculated, and the other is the vaccine. These two parent individuals means:

p1:set out from city 2, and passes the cities of 5, 8, 3, 7, 4, 6 and 1 successfully.

p2:set out from city 5, and passes the cities of 1, 2, 8, 3, 6, 7 and 4 successfully.

The crossover process is described as follows:

 Select two crossover points stochastically (the part which is partitioned by the vertical lines between the two crossover points is called crossover section), then exchange the crossover sections of the parent individuals, that is to say, copy the crossover section of parent individual 1 (p1) to offspring 2 named o2, at the same interval, similarly, the initial part of crossover section of parent 2 (p2) will be copy to the same position of offspring 1 named o1.

 Delete the contents (city number) of p1, which is in the crossover section of p2, and similarly, delete the contents of p2, which belongs to the crossover section of p1.

 Fill the rest part of p2 to o1 behind its second crossover point, if it's over the end of o1 then turn to its head and continue its fill operation until all the rest of p2 were filled to o1, and have the same operation to p1 and o2 to accomplish an order crossover.

For instance, for the parents given above, suppose crossover points showed as follows ("|" denotes crossover points):

p1 = (2 5 | 8 3 7 | 4 6 1)

p2 = (5 1 | 2 8 3 | 6 7 4)

The offspring generated by crossover operation were denoted by o1 and o2, here "#" denotes undetermined cities, and the results of crossover copy operation are:

o1 = (# # | 8 3 7 | # # #)

o2 = (# # | 2 8 3 | # # #)

Fill the rest of the parents to the offspring:

o1 = (6 4 | 8 3 7 | 5 1 2)

o2 = (6 1 | 2 8 3 | 5 7 4)

Select the high fitness valued one from offspring o1 and o2 and replaces the original individual to accomplish the vaccination inoculation.

### Adaptive Mutation Operation

In line 2.1.3, the algorithm selects some solutions obtained by a sub-colony to have mutation operation with the mutation probability of , which is adaptively defined according to the densities of the solutions. This means the sub-colony with higher diversity is more likely to be selected to have mutation. In this formula, m denotes the population size of the sub-colony. The probability for the ant i in the sub-colony to be selected to have mutation is exp(-*δ*). It can be seen that the higher fitness the solution has, the higher probability this solution will be chosen. In mutation operation, if the fitness value between different solutions in the colony is large enough, this indicates a high diversity of the solution, the algorithm would have mutation operation with a low probability, otherwise it will mutate with a high probability to get rid of local convergence and to improve the solution diversity.

### Immune Memory and Immune Metabolism

In the immune system of the organism, in the long period of time after the antigen vanished from the immune system, the cells formed by antibody still memorizes the information of antigen. Consequently, when the antigen invades again, the organism will generate antibodies responsively to counteract the antigen according to its immune network.

In line 2.1.4 of our algorithm, we adopt this mechanism to have immune memory operation so as to increase the quality of the solutions. First, the algorithm calculates the fitness value of each ant in the colony, if the highest one is higher than the current optimal solution, it is added to the memory list, otherwise some better solutions obtained in previous iterations would be selected from the memory list and have crossover and mutation to get another higher fitness valued solution as an antibody.

In the organism, there are about 5% low inspirited cells died every day for its metabolism, and there are also neonatal cells generated by the marrow. Among these neonatal cells only those who have strong adaptability can be integrated into the immune system. By simulating this metabolism behaviour, in the end of each iteration, our algorithm picks out 5% low fitness valued ants and replaces them by the new stochastically generated solutions.

## Authors' contributions

LQ conceived, designed and performed the study under the supervision of LC and YP; LQ designed algorithms, wrote the computer code, collected and analyzed the data; LQ and LC wrote the manuscript; All authors have read and approved the final manuscript.

## Figures and Tables

**Table 1:** The experimental results of symmetric TSP.

Instance	Algorithm	Average solution	Average (s)	Allowed (s)
d198	TA	15781.2	125.4	300
	DGAA	15780.3	117.0	
lin318	TA	42029.7	176.6	500
	DGAA	42029.3	135.4	
pcb442	TA	50789.9	418.2	800
	DGAA	50782.2	399.2	
att532	TA	27686.8	524.8	1000
	DGAA	27686.4	482.5	

The optimal combination of parameters get from lots of test issues is: *ρ *= 0.4, Q = 5, *α *= 1 and *β *= 1. Meanwhile, the number of the ants equals the amounts of the cities, and perform 25 trials (1500 iterations in each trial) on each problem. The experimental results are shown in Table 1, Table 2 and Table 3, where traditional algorithm stands for the MMAS algorithm, average is the average time required to find the best solution in a trial, allowed is the longest time allowed to be executed in each trial, and number means the number of trials to obtain the best solution. The numbers included in the name of the problems denote the numbers of cities for its corresponding problem. For the sake of convenient illustration, Traditional Algorithm [10] was noted as TA.

**Table 2:** The experimental results of asymmetric TSP.

Instance	Algorithm	Average solution	Average (s)	Allowed (s)
ry48p	TA	14428.7	80.2	250
	DGAA	14422.2	42.6	
ft70	TA	38673.9	84.8	300
	DGAA	38673.3	67.2	
kro124p	TA	36239.1	109.5	300
	DGAA	36236.2	72.3	
ftv170	TA	2755.2	121.3	500
	DGAA	2755.0	115.5	

The tables show that the quality of improved algorithm in this paper is much higher than that of TA. It also can be seen from the table that since DGAA reach the optimal solution in much less iterations, it has much higher processing speed, and stronger capability of finding optimal solutions.

**Table 3:** The average iterations.

Instance	TA	DGAA
d198	1759	1235
lin318	2468	1762
ry48p	874	595
ft70	1037	821

As shown in Table 3, the average number of iterations to reach the best solution on d198 using TA is 1650 while the average number is 1221 using DGAA. This fact shows that DGAA saves much more computing time.

## References

[B1] Dorigo M, Maniezzo V (1996). Ant Colony system: Optimization by a colony of coorperating agents. IEEE Transactions on Systems, Man, and Cybernetics-Part B.

[B2] Dorigo M, Gambardella LM (1997). Ant colonies for the traveling salesman problem. BioSystems.

[B3] Gutjahr JW (2000). A Graph-based Ant System and its convergence. Future Generation Computer Systems.

[B4] Talbi EG, Roux O, Fonlupt C, Robillard D (2001). Parallel Ant Colonies for the quadratic assignment problem. Future Generation Computer Systems.

[B5] Maniezzo V, Carbonaro A (2000). An ANTS heuristic for the frequency assignment problem. Future Generation Computer Systems.

[B6] Chang CS, Tian L, Wen FS (1999). A new approach to fault section in power systems using Ant System. Electric Power Systems Research.

[B7] Wu Q, Zhang J, Xu X (1999). An ant colony algorithm with mutation features. Journal of Computer Research & Development.

[B8] Gambardella LM, Dorigo M (1995). Ant-Q: A reinforcement learning approach to the traveling salesman problem. Proceedings of ML-95, the 12th International Conference on Machine Learning.

[B9] Dorigo M, Luca M (2002). The Ant-Q algorithm applied to the nuclear reload problem. Annals of Nuclear Energy.

[B10] Stützle T, Hoos HH (2000). MAX-MIN Ant System. Future Generation Computer Systems Journal.

[B11] Hai W, Yijun L, Yuqiang F, Jianfeng L, Anshi X (2003). Application of artificial immune algorithm in e-commerce oriented negotiation support system. 2003 International Conference on Machine Learning and Cybernetics.

[B12] Guiliang Y, Wu QMJ (2003). The multi-sensor fusion: image registration using artificial immune algorithm. Soft Computing Techniques in Instrumentation, 2003 IEEE International Workshop on Measurement and Related Applications.

[B13] Lin Y, Jumin H, Zhuoshang J, Yinsheng Dai (2000). A study to genetic algorithm based on isolation niche technique. Journal of systems engineering.

